# Changes in Macular Thickness after Cataract Surgery in Patients with Open Angle Glaucoma

**DOI:** 10.3390/diagnostics13020244

**Published:** 2023-01-09

**Authors:** Giedre Pakuliene, Neda Rylskyte, Loreta Kuzmiene, Brent Siesky, Alice Verticchio, Alon Harris, Ingrida Januleviciene

**Affiliations:** 1 Ophthalmology Department, Lithuanian University of Health Sciences, LT-50103 Kaunas, Lithuania; 2 Icahn School of Medicine at Mount Sinai, New York, NY 10029, USA

**Keywords:** cataract, glaucoma, retinal thickness, Optical Coherence Tomography, aqueous humour flare, Retinal Circulation

## Abstract

Background: The purpose of this study was to examine the changes in IOP, total macular and RNFL, ganglion cell layer (GCL) thickness, and aqueous humour flare in open angle glaucoma (OAG) patients before and 6 months after cataract surgery. Methods: This was a prospective observational case–control age- and gender-matched study. Groups: 40 subjects in a controlled OAG (OAG_c_) group, 20 subjects in an uncontrolled OAG (OAG_u_) group, and 60 control group subjects. Examination: complete ophthalmic evaluation, IOP measurement, anterior and posterior segment Optical Coherence Tomography (OCT), and laser flare photometry before and 6 months postoperatively. Results: Six months postoperatively IOP decreased in all groups. An increase in macular thickness was found postoperatively in all groups. Preoperative aqueous humour flare was higher in the OAG_c_ group than in the control group. After cataract surgery, aqueous humour flare was higher in the control group compared to the preoperative result. Conclusions: Changes in IOP following cataract surgery were strongly negatively correlated with preoperative IOP. An increase in macular thickness was observed 6 months postoperatively in all groups. Aqueous humour flare did not differ in OAG_c_ and OAG_u_ groups pre- and postoperatively but significantly increased in the control group postoperatively.

## 1. Introduction

Glaucoma is a multifactorial optic neuropathy characterized by a progressive loss of retinal ganglion cells (RGC) and axons which results in visual field impairment [1,2]. The main goal of glaucoma treatment is to preserve visual function by lowering intraocular pressure (IOP), which is the main modifiable risk factor for glaucoma [3]. Cataract is a condition which presents as clouding of the normally clear lens and decreased visual acuity [4]. Glaucoma is the second-leading cause of blindness in the world after cataracts, and both are age-related ocular comorbidities [5,6]. Open angle glaucoma (OAG) is considered when the anterior chamber angle is proved to be gonioscopically open [1]. The literature suggests surgical treatment for cataract may reduce IOP; however, there is significant variation in the currently available data [7,8,9,10,11,12,13,14,15,16,17,18,19,20,21,22,23,24]. There is still no consensus on what accounts for differences in IOP results and why some patients have a marked reduction in IOP while others do not.

The macula is a vertical oval-shaped area that contains the highest density of RGC—about 30–50% of all RGC are located in the parafoveolar region within a radius of 4.5 mm from the central fovea [25,26]. This is an ideal location for early detection of ganglion cell loss, as RGCs in the macular area make up to 30–35% of the macular thickness [26,27]. In glaucoma patients reduction in macular thickness is caused by the loss of RGCs and their axons, resulting in a decrease in retinal or retinal nerve fibre layer (RNFL) [26,27]. Inflammatory mediators released during cataract surgery are known to increase perifoveal capillary permeability, leading to increased macular thickness and sometimes cystoid macular edema [28].

Aging is known to affect the human vasculature throughout the body and it is known that retinal blood flow decreases with age [29]. In patients with glaucoma, a decrease in macular vascular density correlates with peripapillary RNFL thinning in the corresponding quadrants [30] as well as with central macular defects [31]. This is a clinically relevant sign that can be used to follow the progression of open angle glaucoma. Optical Coherence Tomography Angiography (OCT-A) can detect a focal decrease in vascular density in the parafoveolar region [31,32]. The greater the glaucomatous lesion (higher stage), the lower the macular vascular density [33,34,35]. The literature indicates that functional retinal hyperemia and increased blood flow can be observed 3 months after cataract surgery, which is explained as the result of increased retinal photostimulation [36].

Laser flare photometry is a non-invasive and objective method for evaluating ocular inflammation by quantifying biomarkers of inflammation within the anterior chamber fluid [37]. The results of laser flare photometry are low in healthy individuals with no history of eye disease and eye surgery [38], while a rise in aqueous humour flare is caused by an increased protein concentration in the aqueous humour due to increased permeability of the blood–aqueous barrier [37,39,40,41,42]. In practice, laser flare photometry is usually used to monitor the course and severity of uveitis and endophthalmitis [43,44]. During intraocular inflammation, the numerical value of the aqueous humour flare can increase three to four times in the case of mild inflammation, and several tens of times in the case of HLA-27-related anterior uveitis [44]. In addition, there are data reporting an increase in aqueous humour flare after cataract surgery [28].

The purpose of this study was to examine changes in IOP, total thickness of the macula, thickness of RNFL, ganglion cell layer ++ (GCL++), ganglion cell + (GCL+) and choroid, foveal avascular zone (FAZ) parameters, and aqueous humour flare in OAG patients before and 6 months after minimally invasive cataract surgery.

## 2. Materials and Methods

This prospective observational case–control age- and gender-matched study was conducted at the Lithuanian University of Health Sciences, Kaunas, Lithuania. The Kaunas Regional Biomedical Ethics Committee approved all study procedures and participants signed an informed consent prior to participation.

Inclusion criteria: subjects aged >40 years and scheduled for cataract phacoemulsification surgery with intraocular lens (IOL) implantation, best corrected visual acuity (BCVA) >0.2 (Snellen chart), and lens opacification not blocking view of the fundus.

Exclusion criteria: closed angle or congenital glaucoma, other pathologies of anterior and posterior segment of the eye (e.g., age-related macular degeneration, preretinal fibrosis, optic nerve pathologies, congenital eye pathologies, corneal pathology, blocking the view of the fundus), ocular and orbital trauma, intraocular surgery in the past (trabeculectomy, canaloplasty, corneal transplantation, vitrectomy), refractive surgery, laser procedures (laser trabeculoplasty, laser cyclodestruction), systemic uncontrolled conditions, pregnant or breastfeeding women.

There were three groups of participants:

1. Controlled IOP OAG (OAG_c_) group: patients diagnosed with cataract and OAG with IOP <21 mmHg and treated with medical antiglaucomatous treatment for more than 2 years.
2. Uncontrolled IOP OAG (OAG_u_) group: patients diagnosed with cataract and OAG (proved gonioscopically) with IOP >21 mm Hg and treated with medical antiglaucomatous treatment for more than 2 years.
3. Control group: patients diagnosed with cataract and without previous OAG diagnosis.

Subjects were examined twice: at baseline and 6 months after the cataract surgery. At each visit patients received a complete ophthalmic evaluation including IOP via Goldmann tonometery, anterior and posterior segment Optical Coherence Tomography (OCT) and OCT-A of the macula (DRI OCT Triton plus ver. 10.13), and evaluation of laser flare photometry (Laser Flare Meter Kowa FM-700 ver. 2.01.200000, Kowa, Japan).

Aqueous humour flare was analyzed without pupil dilation [40,45]. Ten measurements were obtained from each eye and marginal values were eliminated to increase accuracy. Flare count was presented as photon count per millisecond (pc/ms).

**Figure 1:**
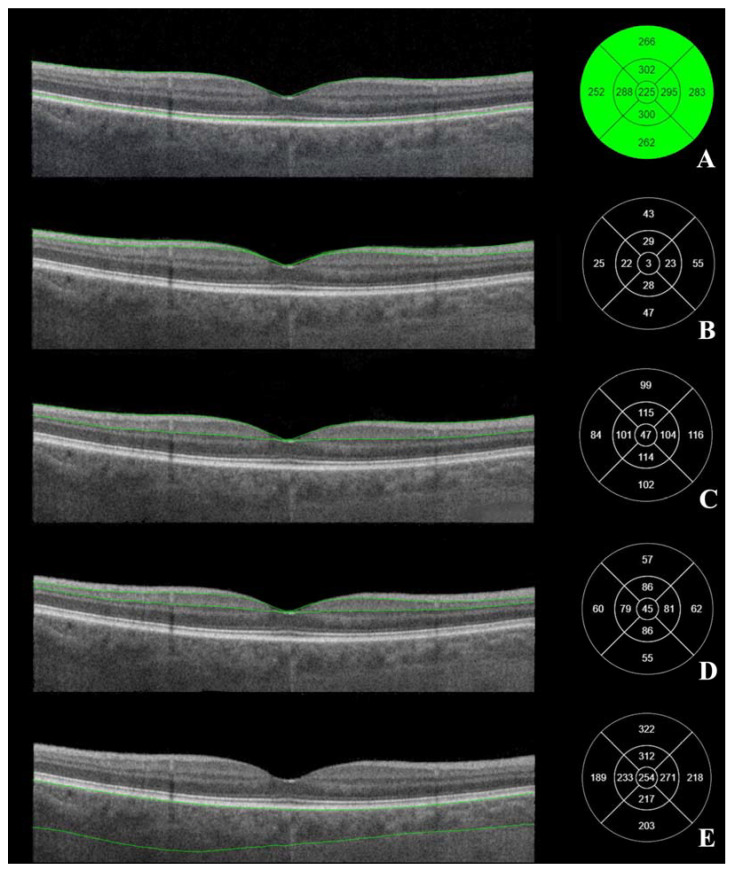
Macular OCT. Macular layers marked on the right. (**A**) the total thickness of the macula, (**B**) retinal nerve fiber layer (from internal limiting membrane to the interface between retinal nerve fiber layer and ganglion cell layer), (**C**) GCL++ layer (from internal limiting membrane to the interface between inner plexiform layer), (**D**) GCL+ layer (from the interface between retinal nerve fiber layer and ganglion cell layer to the interface between inner plexiform layer and inner nuclear layer). (**E**) choroid thickness (from retinal pigment epithelium to the interface between choroid and sclera). Left—ETDRS–9 segments and thickness of the measured layer (µm). (Images were acquired with DRI OCT Triton plus (Ver.10.13). Final image was finished using ImageJ program and Clip Studio Paint PRO program Ver. 1.9.10 ©CELSYS Inc. https://www.clipstudio.net/en/).

Posterior segment OCT was performed in semi-dark conditions with a seated subject with dilated pupils (Tropicamide 10 mg/mL drops). The macular OCT scan type was “3D Macula V/H”, which was a 7 × 7 mm cube scan and allowed us to automatically outline the RNFL and GCL+ complex (Figure 1).

The cowest accepted quality of OCT margin was 40/100 quality points. Macular tomograms were analyzed using the Early Treatment Diabetic Retinopathy Study (ETDRS–9) grid at 9 different subfields of macula. The thickness (μm) of RNFL, GCL+, GCL++, the total macular thickness, and the choroid thickness were measured automatically in all 9 subfields of ETDRS–9 (Figure 1). The central subfield was at a 1 mm diameter circle around the central foveal point; at a 3 mm diameter circle were the superior, temporal, inferior and nasal inner subfields; at a 6 mm diameter circle were the superior, temporal, inferior and nasal outer subfields.

**Figure 2:**
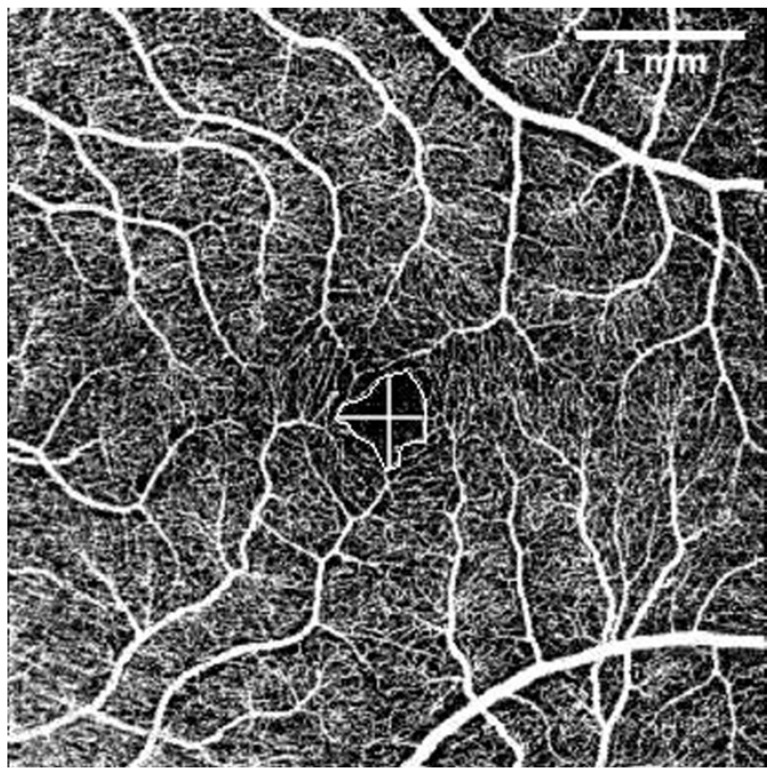
Superficial capillary layer OCT-A. FAZ perimeter, horizontal, and vertical diameters. The intraobserver repeatability of 30 randomly chosen tomograms was excellent (Pearson’s correlation coefficient (PCC) = 0.9). The scale was 71.11 pixels/mm; knowing the size of the tomogram (4.5 × 4.5 mm, 320 × 320 pixels), the FAZ area was measured by connecting the terminal points of vessels that form the FAZ. Horizontal and vertical diameters were measured at the largest points of the FAZ horizontally and vertically, respectively. Terminal vessel counts were the number of terminal vessels that formed the vessel circle around the FAZ. (Images were acquired with DRI OCT Triton plus (Ver.10.13). Final image was finished using ImageJ program and Clip Studio Paint PRO program Ver. 1.9.10 ©CELSYS Inc. https://www.clipstudio.net/en/).

The OCT-A scans were performed under the same conditions as the posterior segment OCT and were repeated until obtained without eye movement or blinking artefacts. For evaluation of OCT-A images the Fiji program package (Version 2.1.0/1.53c) was used [46]. Foveal Avascular Zone (FAZ) area (mm^2^), perimeter (mm), vertical and horizontal diameter (mm), and the total count of terminal macular vessels in OCT-A was manually measured by one examiner (G.P.) (Figure 2).

All of the participants underwent an uneventful cataract surgery by the same surgeon. Surgical technique included temporal incision in clear cornea, capsulorhexis of 5.5 mm diameter, hydrodissection, lens nucleus phacoemulsification, cortical mass removal using irrigation and aspiration, implantation of intraocular lens (Tecnis® 1 monofocal IOL Model ZCB00 (Johnson and Johnson Vision, USA), Alcon AcrySof® IQ IOL (Alcon, Switzerland and USA), and EnVista®IOL (Bausch + Lomb, USA).

Statistical analysis was performed using the IBM SPSS Statistics program package (Armonk, NY, USA: IBM Corp, ver. 23.0). Student’s T-test and Bonferroni criterion were used to compare normally distributed independent samples, Mann–Whitney U and Kruskal–Wallis tests for nonparametric independent samples, and Spearman’s rho correlation coefficient for rank-order correlations in nonparametric samples. Fisher’s exact test was used to compare two independent small samples (2 × 2). The Wilcoxon test was used for two dependent samples. McNemar’s test was used for two paired nominal samples. *P* < 0.05 was considered statistically significant.

## 3. Results

Demographic data are presented in Table 1: 120 subjects of Caucasian ethnicity were included. A control group of 60 (50%) subjects, OAG_c_ group of 40 (33.3%) subjects, and OAG_u_ group of 20 (16.7%) subjects were included.

The matching ratio of the participants was 3:2:1 in the control group, the OAG_c_, and the OAG_u_ groups, respectively. The groups were matched according to age and gender; the age was normally distributed. The mean (SD) BCVA was 0.47 (0.23) in the control group, 0.44 (0.27) in the OAG_c_ group, and 0.54 (0.24) in the OAG_u_ group (*p* = 0.240).

The subjects of the control group did not use any anti-glaucoma eyedrops; the anti-glaucoma drops used by the other study participants are presented in Table 2.

OAG_u_ group subjects received statistically significantly more different antiglaucomatous compounds compared to the OAG_c_ group. OAG_u_ group subjects were prescribed statistically significantly more different bottles of medications compared to the OAG_c_ group. Although prostaglandin analogues were found to be administered equally frequently in both groups, carbonic anhydrase inhibitors, beta blockers, and alpha-2 agonists were used more often in the OAG_u_ group.

### 3.1. IOP before and Six Months after the Cataract Surgery

IOP was measured before and 6 months after cataract surgery. Results are shown in Table 3.

**Figure 3:**
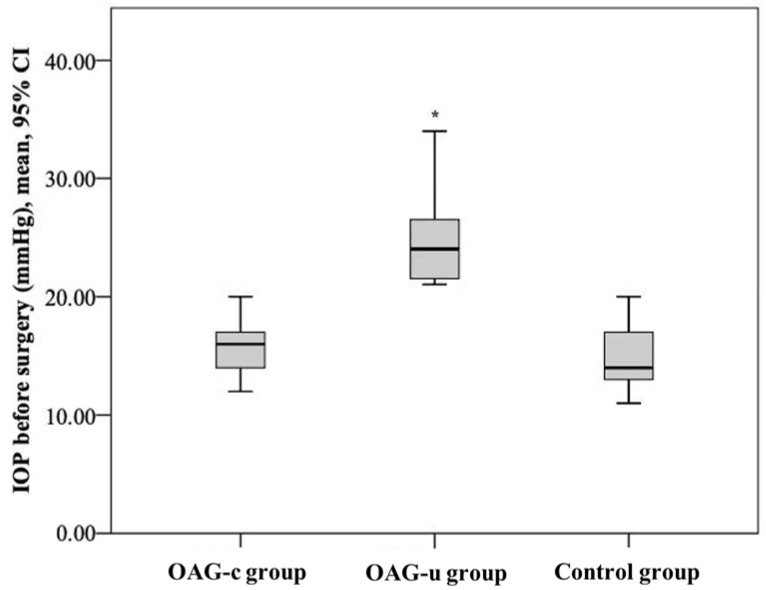
IOP before cataract surgery in all groups. Asterisk (*) marks statistically significantly higher IOP (*p* < 0.001).

IOP before cataract surgery did not differ between the control group and the OAG_c_ group (*p* = 0.322), but it was statistically significantly lower than in the OAG_u_ group (*p* < 0.001). Even though IOP did not differ between the control group and the OAG_c_ group, a tendency of lower IOP was found in the control group. IOP in the OAG_c_ group was statistically significantly lower than in the OAG_u_ group (*p* < 0.001) (Figure 3).

**Figure 4:**
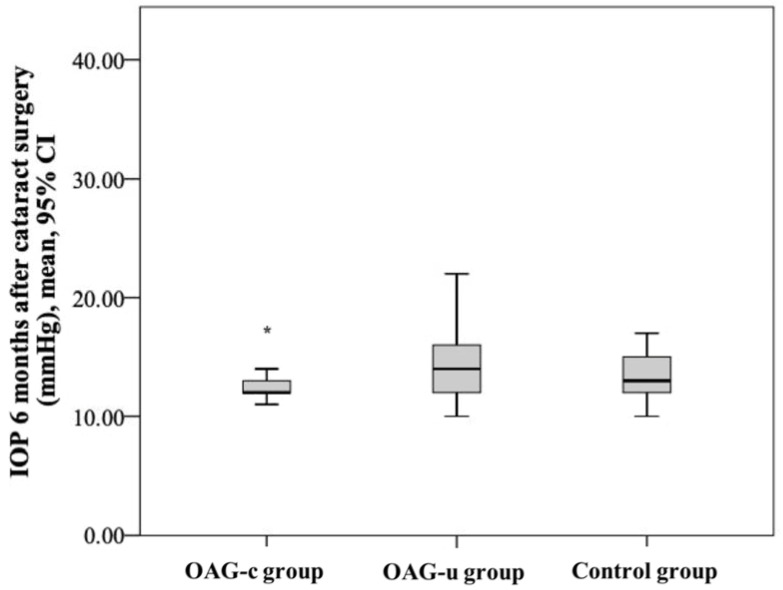
IOP 6 months after cataract surgery in all groups. Asterisk (*) marks statistically significantly lower IOP in the OAG_c_ group than in the OAG_u_ group (*p* < 0.001).

IOP six months after cataract surgery was statistically significantly lower in the OAG_c_ group than in the OAG_u_ group (*p* = 0.039). IOP after surgery did not differ between both the control group and the OAG_c_ group and the control group and the OAG_u_ group (*p* > 0.05 and *p* > 0.05, respectively) (Figure 4).

**Figure 5:**
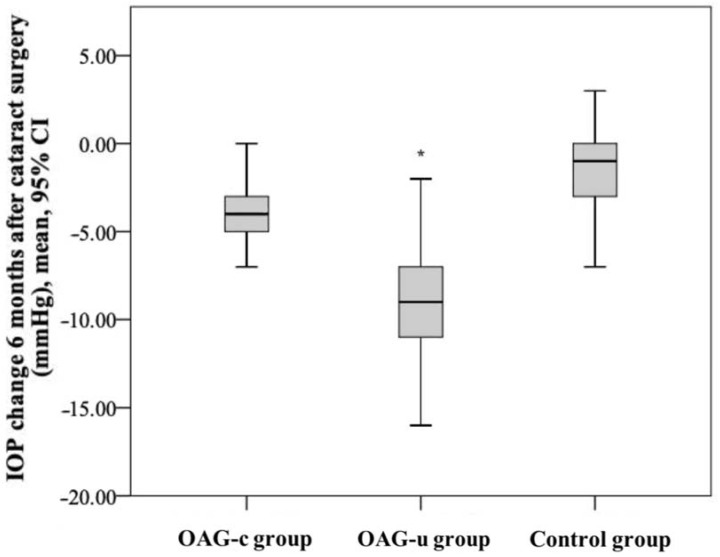
IOP change 6 months after cataract surgery in all groups. The highest negative IOP change was found in the OAG_u_ group (*) (*p* = 0.018).

IOP reduction in the control group was statistically significantly lower than in the OAG_c_ group (*p* = 0.001). IOP reduction in the OAG_u_ group was statistically significantly higher than in the OAG_c_ group (*p* = 0.018). IOP reduction in the OAG_u_ group was statistically significantly higher than in the control group (*p* < 0.001) (Figure 5).

**Figure 6:**
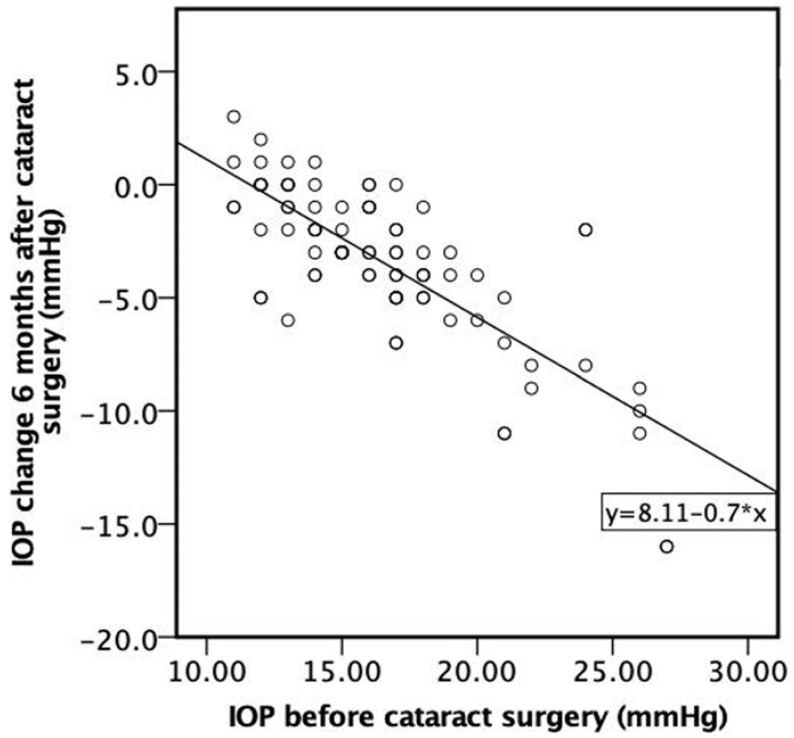
Correlation between IOP before cataract surgery and IOP change 6 months after cataract surgery.

A strong negative correlation was found between IOP before cataract surgery and IOP change 6 months after cataract surgery. The higher the IOP before cataract surgery, the higher the negative IOP change 6 months following cataract surgery (ρ = −0.706, *p* < 0.001) (Figure 6).

### 3.2. Posterior Segment OCT Results before the Cataract Surgery

**Figure 7:**
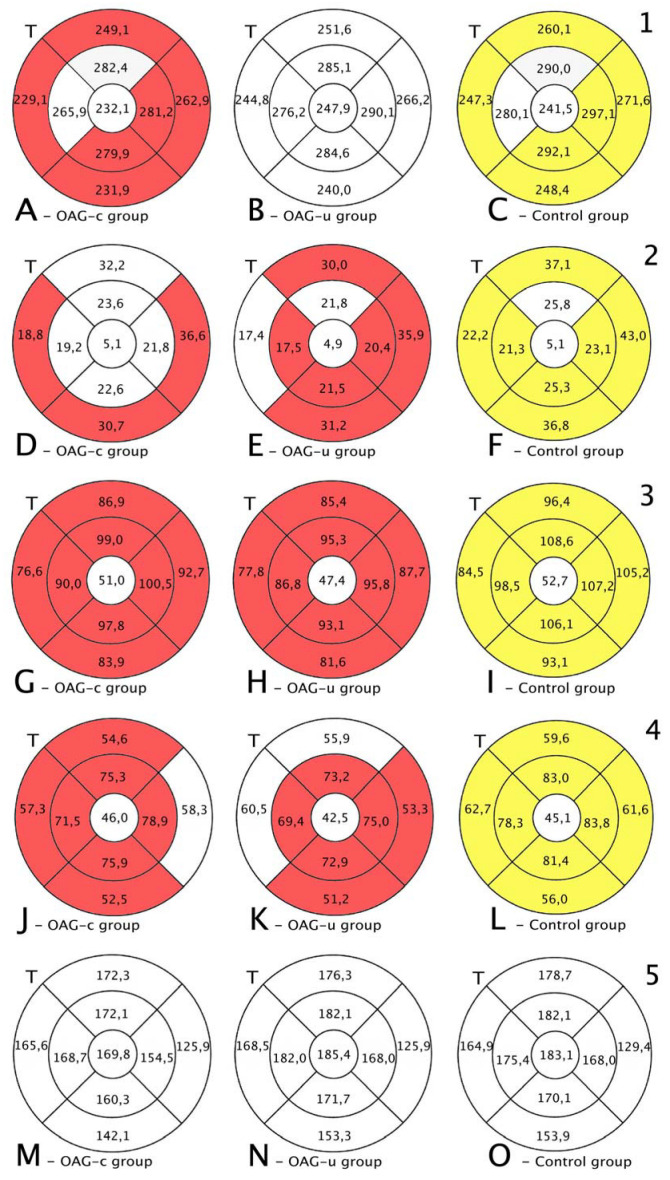
Comparison of posterior segment OCT results in ETDRS–9 subsegments and retinal layers before the cataract surgery between control and OAG_c_ or OAG_u_ groups. (**A**–**C**) total macular thickness, (**D**–**F**) RNFL thickness, (**G**–**I**) GCL++ thickness, (**J**–**L**) GCL+ thickness, (**M**–**O**) choroid thickness. Temporal side (T) is shown on the left. Mean layer thickness is shown in ETDRS–9 subsegments. Colours mark statistically significant differences: yellow—statistically significantly higher mean thickness, red—statistically significantly lower mean thickness, white—statistically significant difference not found.

Total macular thickness was statistically significantly lower in the OAG_c_ group than in the control group in all subsegments except the central, the inner superior, and the inner temporal subsegments (*p* < 0.05). There were no statistically significant differences in total macula thickness in all subsegments in the OAG_u_ group compared to the control group (Figure 7, first row).

RNFL was statistically significantly lower in the OAG_u_ group compared to the control group in all subsegments except the inner superior subsegment (*p* < 0.05). RNFL in the OAG_c_ group did not differ statistically significantly in comparison with the control group in all remaining subsegments except the outer temporal, the outer inferior, and the outer nasal subsegments (Figure 7, second row).

**Figure 8:**
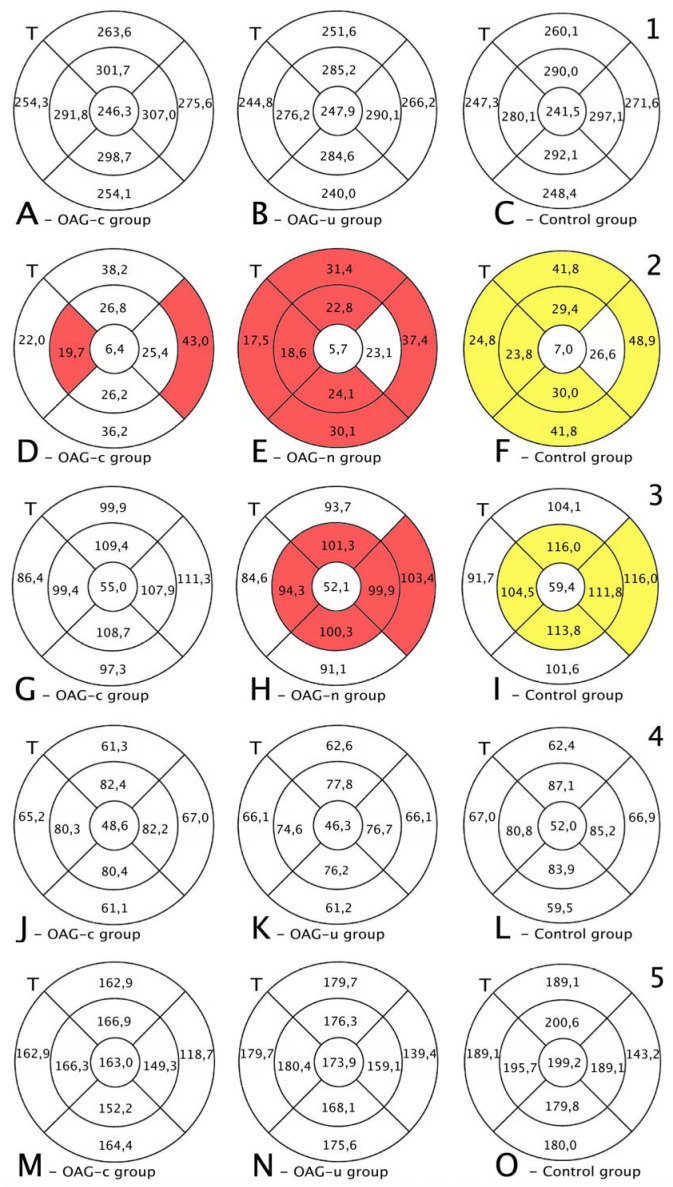
Comparison of posterior segment OCT results in ETDRS–9 subsegments and retinal layers 6 months after the cataract surgery between control and OAG_c_ or OAG_u_ groups. (**A**–**C**) total macular thickness, (**D**–**F**) RNFL thickness, (**G**–**I**) GCL++ thickness, (**J**–**L**) GCL+ thickness, (**M**–**O**) choroid thickness. Temporal side (T) is shown on the left. Mean layer thickness is shown in ETDRS–9 subsegments. Colours mark statistically significant differences: yellow—statistically significantly higher mean thickness, red—statistically significantly lower mean thickness ean, white—statistically significant difference not found.

GCL++ was statistically significantly higher in all ETDRS–9 subsegments in the control group compared to the OAG_c_ and OAG_u_ groups (*p* < 0.05). The GCL++ layer in OAG_c_ and OAG_u_ groups was not statistically significantly different in all subsegments (*p* > 0.05) (Figure 8, third row).

The GCL+ thickness was statistically significantly lower in all four inner subsegments in the OAG_c_ and OAG_u_ groups compared to the control group (*p* < 0.05). The GCL+ layer was statistically significantly lower in the OAG_c_ group compared to the control group in the outer superior, the outer temporal, and the outer nasal subsegments (*p* < 0.05). The GCL+ layer was statistically significantly lower in the OAG_u_ group in comparison with the control group in the outer inferior and the outer nasal subsegments (*p* < 0.05) (Figure 8, fourth row).

Choroidal thickness did not differ significantly in all ETDRS–9 subsegments in all groups (Figure 7, fifth row).

### 3.3. Posterior Segment OCT Results Six Months after the Cataract Surgery

**Figure 9:**
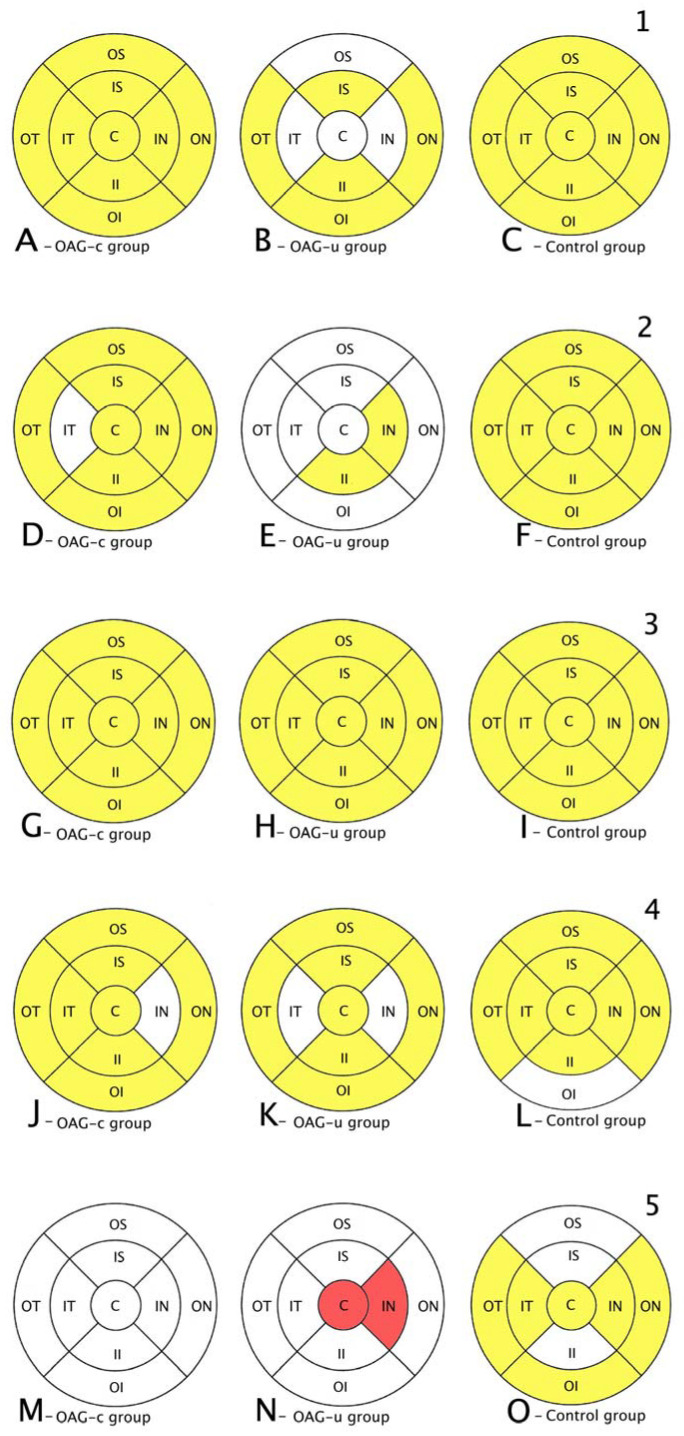
Comparison of posterior segment OCT results in ETDRS–9 subsegments and retinal layers before and 6 months after the cataract surgery between control and OAG_c_ or OAG_u_ groups. (**A**–**C**) total macular thickness, (**D**–**F**) RNFL thickness, (**G**–**I**) GCL++ thickness, (**J**–**L**) GCL+ thickness, (**M**–**O**) choroid thickness. C—central, IS—inner superior, IN—inner nasal, II—inner inferior, IT—inner temporal, OS—outer superior, ON—outer nasal, OI—outer inferior, OT—outer temporal. Mean layer thickness is shown in ETDRS–9 subsegments. Colours mark statistically significant differences: yellow—statistically significantly higher mean thickness, red—statistically significantly lower mean thickness, white—statistically significant difference not found.

The total thickness of the macula did not differ among all groups (*p* > 0.05) (Figure 9, first row).

RNFL was statistically significantly lower in the OAG_u_ group compared to the control group in all subsegments, except the central and inner nasal subsegments. RNFL was statistically significantly lower in the inner temporal and outer nasal subsegments in the OAG_c_ group compared to the control group (Figure 8, second row).

GCL++ was statistically significantly lower in the OAG_u_ group than in the control group in the inner superior, inner temporal, inner nasal, and outer nasal subsegments. GCL++ did not statistically significantly differ between the OAG_c_ and control groups (*p* > 0.05) (Figure 8, third row).

GCL+ and choroidal thickness did not differ significantly in all ETDRS–9 subsegments in all groups (Figure 8, fourth and fifth rows).

### 3.4. Comparison of Posterior Segment OCT Results before and Six Months after the Cataract Surgery

The total thickness of the macula increased in all subsegments in OAG_c_ and control groups, while in the OAG_u_ group an increase in the total macula thickness was found only in the inner superior, inner inferior, outer temporal, and outer nasal subsegments (Figure 9, first row).

An increase in RNFL thickness was found in the OAG_c_ group in all subsegments, except the inner temporal subsegment, whereas in the OAG_u_ group an increase in RNFL thickness was observed only in the inner nasal and temporal subsegments. RNFL thickness increased in all subsegments in the control group (Figure 9, second row).

A GCL++ thickness increase was found in all subsegments in all groups (Figure 9, third row). A GCL+ thickness increase was found in the OAG_c_ group in all subsegments, except the inner nasal subsegment. In the OAG_u_ group the GCL+ thickness increased in all subsegments, except the inner nasal and the inner temporal subsegments. A GCL+ thickness increase was found in the control group in all subsegments, except the outer inferior subsegment (Figure 9, fourth row).

Choroid thickness did not change significantly in the OAG_c_ group. In the OAG_u_ group choroid thickness decreased in the central and inner nasal subsegments. Choroid thickness increased in the control group in all subsegments, except the outer and inner superior and the inner inferior subsegments (Figure 9, fifth row).

### 3.5. Comparison of Macula OCT-A Results before and Six Months after the Cataract Surgery

**Figure 10:**
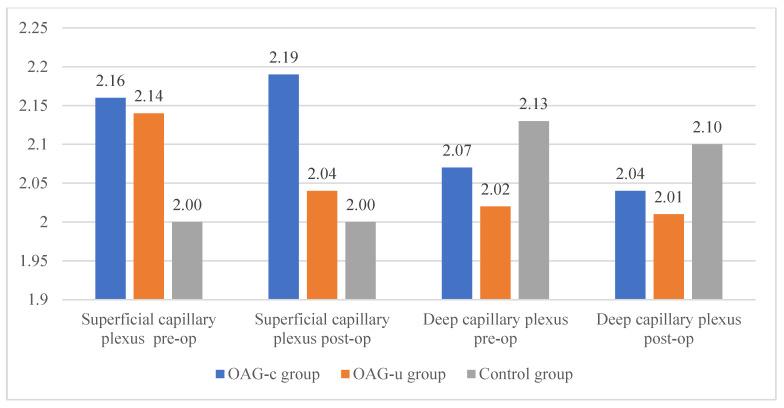
FAZ perimeter of superficial and deep capillary layers before and 6 months after cataract surgery. Vertical axis shows FAZ perimeter (mm).

Statistically significant differences in the FAZ perimeter of the superficial or deep capillary plexuses were not observed in any of the study groups. The FAZ perimeter did not differ preoperatively and postoperatively in all groups (Figure 10).

**Figure 11:**
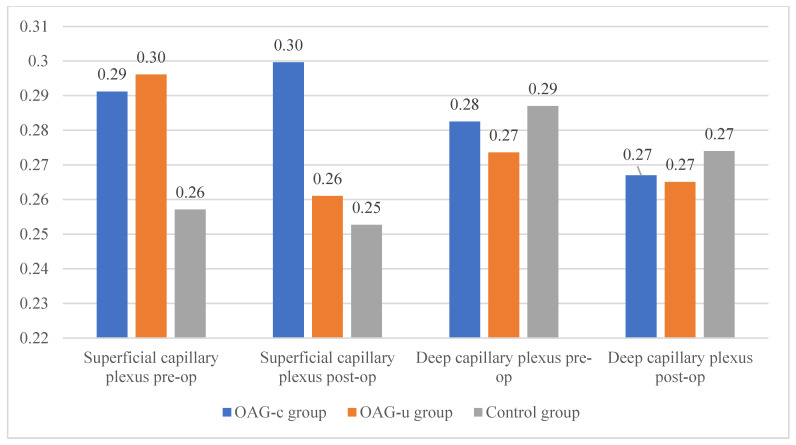
FAZ area of superficial and deep capillary layers before and 6 months after cataract surgery. Vertical axis shows FAZ area (mm^2^).

There were no statistically significant FAZ area differences in any of the groups in the superficial or deep capillary plexuses. In addition, the FAZ area did not differ pre- and postoperatively in all groups (Figure 11).

**Figure 12:**
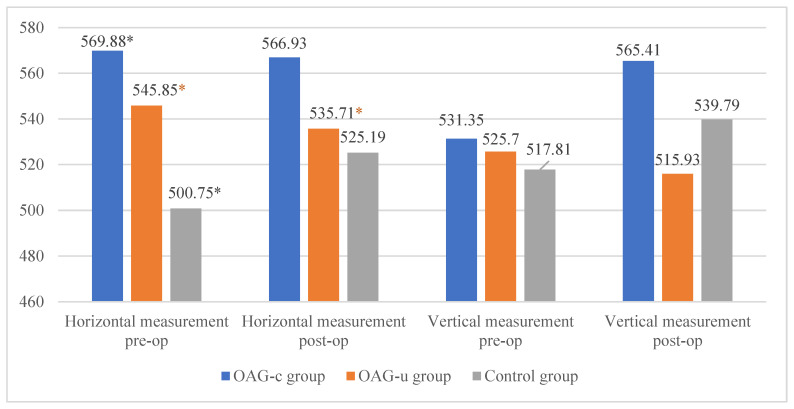
FAZ horizontal and vertical measurements of superficial capillary plexus before and 6 months after cataract surgery. Vertical axis shows mean FAZ horizontal and vertical measurements (μm). Statistically significant measurements (*) (*p* < 0.05).

The horizontal measurement of the superficial capillary plexus before cataract surgery was statistically significantly higher in the OAG_c_ group compared to the control group (*p* = 0.009). In the OAG_u_ group the horizontal measurement before the surgery was statistically significantly higher than 6 months after surgery (*p* = 0.030). The vertical measurement before the surgery did not differ statistically significantly in all groups (*p* > 0.05). In the OAG_c_ group the vertical measurement was statistically significantly lower after the surgery than before the surgery (*p* = 0.008) (Figure 12).

**Figure 13:**
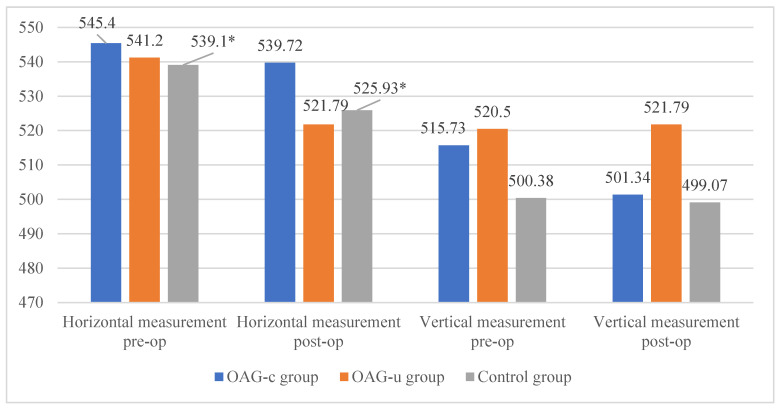
FAZ horizontal and vertical measurements of deep capillary plexus before and 6 months after cataract surgery. Vertical axis shows mean FAZ horizontal and vertical measurements (μm). Statistically significant measurements (*) (*p* < 0.05).

There were no statistically significant differences in FAZ horizontal and vertical measurements of the deep capillary plexus before and after the cataract surgery in all study groups (*p* > 0.05 and *p* > 0.05). The horizontal measurement of control group subjects was statistically significantly higher before the surgery than 6 months after the surgery (*p* = 0.030) (Figure 13).

### 3.6. Laser Flare Photometry before and Six Months after Cataract Surgery

**Figure 14:**
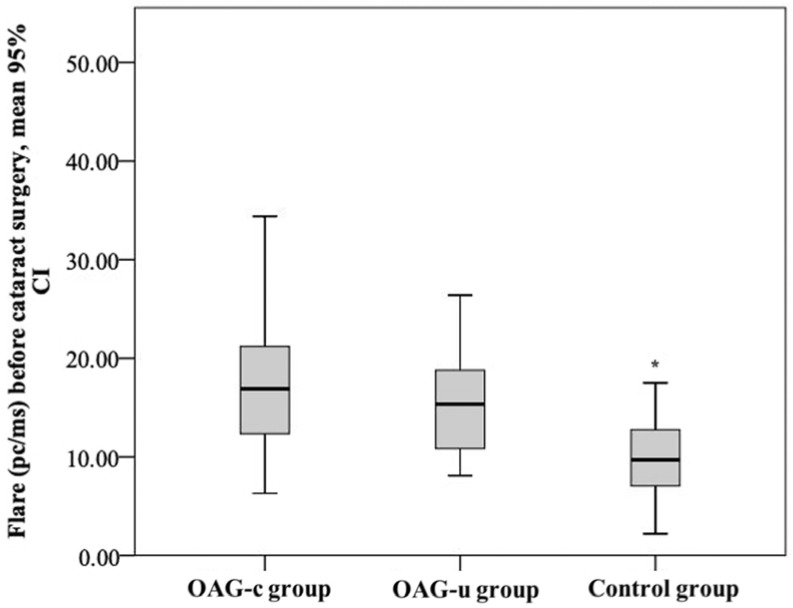
Aqueous humour flare before cataract surgery in all groups. Aqueous humour flare was statistically significantly lower in the control group (*) (*p* < 0.001).

The aqueous humour flare mean (SD) before cataract surgery was 18.52 (9.0) pc/ms in the OAG_c_ group, 15.8 (5.5) pc/ms in the OAG_u_ group, and 10.15 (4.5) pc/ms in the control group. The aqueous humour flare mean in the control group was statistically significantly lower than in the OAG_c_ group and the OAG_u_ group (*p* < 0.001). The mean of the aqueous humour flare did not differ significantly between the OAG_c_ group and the OAG_u_ group (*p* = 0.779) (Figure 14).

**Figure 15:**
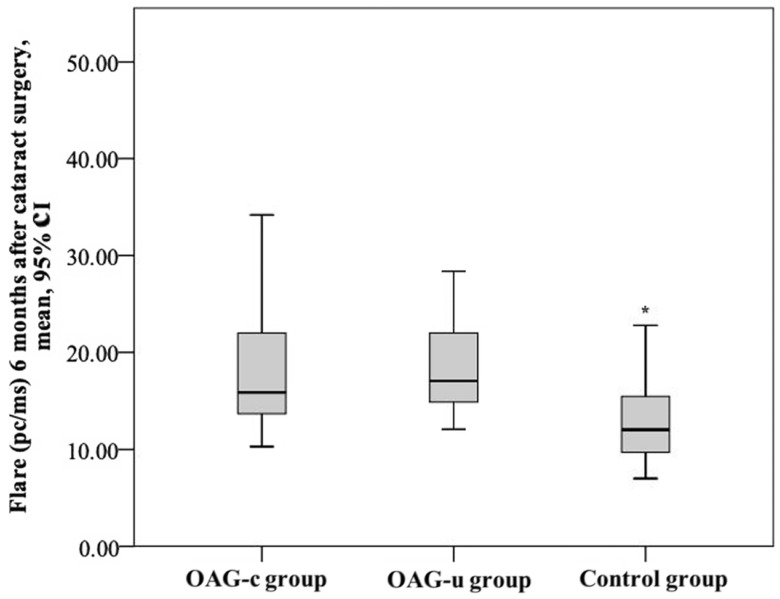
Aqueous humour flare 6 months after cataract surgery in all groups. Asterisk (*) marks statistically significantly lower mean aqueous humour flare (*p* < 0.001).

After cataract surgery, the aqueous humour flare mean (SD) was 19.5 (10.6) pc/ms in the OAG_c_ group, 17.8 (4.7) pc/ms in the OAG_u_ group, and 12.69 (4.0) pc/ms in the control group. The aqueous humour flare mean in the control group was statistically significantly lower than in the OAG_c_ group and the OAG_u_ group (*p* < 0.001). The aqueous humour flare did not differ significantly between the OAG_c_ group and the OAG_u_ group (*p* = 0.770) (Figure 15).

The mean aqueous humour flare in the control group was statistically lower before cataract surgery than 6 months after cataract surgery (*p* = 0.004). There was no statistically significant aqueous humour flare mean difference between the subjects in the OAG_c_ and OAG_u_ groups before and after surgery (*p* > 0.05).

## 4. Discussion

In this present study we aimed to evaluate the changes in the IOP, total macular and RNFL thickness, ganglion cell layer, choroid, and FAZ parameters, and aqueous humour flare in OAG patients before and 6 months following cataract surgery.

In this study the IOP mean decreased in all study groups 6 months after cataract surgery. The literature shows that cataract surgery has a benefit on lowering the IOP in patients with co-morbid glaucoma [47,48,49,50,51]. OAG patients had a higher IOP drop in comparison with the participants without the OAG. The highest negative IOP change was found in the OAG_u_ group (−8.93 (4.2) mmHg). Iancu et al. [11] found that the higher the IOP before cataract surgery (27–28 mmHg), the higher the negative change could be expected after surgery. We identified a strong negative correlation between IOP change and preoperative IOP in all groups. The higher the IOP before cataract surgery, the higher the negative IOP change was found 6 months postoperatively. It is important to mention that preoperative IOP did not differ significantly between the control and OAG_c_ groups. However, postoperatively, a higher negative IOP change was found in the OAG_c_ group than in the control group. The exact mechanism that explains the decrease in IOP after cataract surgery is still unknown, but probable mechanisms include changes in molecular (effects on the trabecular meshwork), physiological (effects on the ciliary body), and biomechanical (changes in anterior segment anatomy, lens position, fluidics) levels [51]. Factors influencing IOP change are still to be explored. Majstruk et al. [9] found that the IOP for OAG patients can change from +5 mmHg to −5 mmHg one year after cataract surgery. Our analysis showed that all of the participants had an IOP drop after cataract surgery, and it is possible that anterior chamber parameters might be influencing these observed changes.

There is no current universally accepted database for evaluating normal values of macular thickness; however, macular asymmetry is often evaluated and compared with repeated examinations of the same patient in everyday ophthalmological practice [52]. In glaucoma, retinal ganglion cell death and degeneration of the RNFL are detected, resulting in a thinning of the total macular thickness due to multi-layer changes. Dead cells are replaced by glial cells as the main nervous system response to injury (proliferation of astroglia, hypertrophy of cell body, thickening of cell processes) [53]. According to the literature IOP increases during cataract surgery, and episodic macular thickening is observed after cataract surgery [54]. This depends on the duration of the operation [54]. Elevated IOP after cataract surgery increases the risk of cystic macular edema development [55]. Cystic macular edema usually occurs 3–4 weeks postoperatively and up to 3–6 months postoperatively [56]. Yoon and colleagues [57] found that cystic macular edema can cause a wide spectrum of changes ranging from asymptomatic macular cysts that do not deform the contour of the macula and do not affect visual acuity (microcystic macular edema) to cysts deforming the contour of the macula and weakening visual acuity.

In our analysis we did not find cystic macular edema 6 months after uneventful cataract surgery; however, we identified an increase in macular thickness 6 months postoperatively in all groups. A uniform increase in macular thickness was observed in the OAG_c_ and control groups. In the OAG_u_ group, the increase in macular thickness was uneven and differed among ETDRS–9 subsegments. Wang et al. [58] found that macular thickening may be related to the parafoveolar vascular density of the deep retinal vascular layer, especially in diabetes mellitus or complicated cataract surgery. Topical non-steroidal anti-inflammatory drugs (NSAIDs), such as Bromfenac or Nepafenac, reduce the risk of macular edema after cataract surgery [57]. In our study, topical non-steroidal anti-inflammatory drugs were not prescribed after cataract surgery. All patients were prescribed a combination of dexamethasone and chloramphenicol eye drops (1 mg/2 mg/g). Dexamethasone drops also prevent macular edema but to a lesser extent than NSAIDs [59]. Pukl et al. [60] detected a macular thickness increase without a cystic component 6 months after cataract surgery. The results of our study did not contradict Pukl’s findings. Moreover, we found that macular thickness increase can be found in both the RNFL and the ganglion cell layer. The exact mechanism is still not known.

The thickness of the choroid is age-related and on average it decreases 3 μm per year [61]. Choroidal thickness also depends on the general condition of the body, e.g., in hemodialysis patients, choroidal thickness is higher before than after hemodialysis [62]. IOP has also been found to decrease after hemodialysis [62]. Choroidal perfusion depends on the body’s hydration, as well as IOP [63,64]. Ziwei Ma et al. [65] detected that with an increase in IOP by 10 mm Hg, choroid perfusion decreased. In our study, choroid thickness increased in the control group 6 months after cataract surgery. In the OAG_c_ group, a choroid thickness change was not found. In the OAG_u_ group, choroid thickness decreased in the central and inner nasal quadrants. Choroid thickness did not change in the rest of the ETDRS–9 subsegments. The uneven change in choroid thickness could be explained by segmental choroid autoregulation. Gudauskienė and co-authors [66] found that after uneventful cataract surgery, choroid thickness increased episodically and returned to its previous thickness within 3 months. Chen et al. [67] also found episodic choroid thickness increase after cataract surgery (up to 3 months). The literature shows that choroid thickness and blood flow can be found to increase after trabeculectomy [68,69,70]. Different brands of IOL were used in this study. One type of IOL had a blue light filter; however, the sample was too small to compare different types of IOL. Blue-light-blocking IOLs were initially designed to postpone AMD progression and were proven to have little to no effect on vision and macula [71]. However, it could be an interesting topic for future research in glaucoma patients. 

In this study, the FAZ perimeter and area did not differ statistically significantly between any group preoperatively or 6 months postoperatively. The horizontal FAZ measurement before cataract surgery was higher in subjects of the OAG_c_ group compared to control subjects while the horizontal measurement was also significantly higher in the OAG_u_ group before cataract surgery compared to data 6 months after cataract surgery. According to the literature, both FAZ and IOP (>10 mmHg) can decrease after trabeculectomy [72]. In our study, the mean IOP reduction in the OAG_u_ group was 8.93 mmHg; however, a significant IOP decrease did not affect FAZ size. Shiihara et al. [73] found that mean the FAZ perimeter of the superficial capillary layer was 2.278 ± 0.418 mm in healthy subjects. In our study, the FAZ perimeter was similar to Shiihara et al.’s findings, even though we included older patients in our study. According to Ghassemi and colleagues [74] the FAZ area of the superficial capillary plexus was 0.23–0.32 mm^2^ in healthy subjects and the area of the superficial capillary plexus of women is larger than that of men. They also found that the FAZ area of the deep capillary plexus was 0.31–0.40 mm^2^ in healthy subjects and the FAZ area was larger in women than in men [74]. The results of our study did not contradict Ghassemi et al.’s findings, even though we included older patients. It should be kept in mind that a reference database has not yet been created and small differences may occur because of the usage of different OCT-A devices. A larger FAZ perimeter is associated with a larger FAZ area, but also with lower symmetry. The FAZ diameter usually decreases in the final glaucoma stages [75]. We included mostly early-stage glaucoma patients in our study. This could be the main reason why the differences in the FAZ area and perimeter were not observed. FAZ changes were observed although they were not pronounced. The horizontal diameter of the superficial capillary plexus was significantly higher in the OAG_u_ group compared to the control group preoperatively. The same horizontal diameter was significantly higher preoperatively than 6 months postoperatively. This is the same area where we found choroid thickness decrease after surgery, thus these changes might be associated with vascular remodeling of the tissue following a decrease in postoperative IOP.

Aqueous humour flare is considered to be one of the inflammatory parameters of the anterior chamber and intraocular inflammation [39,76]. Aqueous humour flare increases modestly with age [40,42,77], but in this study subjects were age-matched, so this factor did not have a significant effect on the results. Aqueous humour flare also rises in non-inflammatory eye conditions, e.g., pseudoexfoliation syndrome, as well as after intraocular surgeries [78,79,80,81]. On the first day after cataract surgery, aqueous humour flare can rise up to 20–30 pc/ms [80,82]. De Maria et al. [80] also found that aqueous humour flare increases on the first day after cataract surgery and then gradually decreases but still does not reach the preoperative result 6 months later. The authors explain this phenomenon as persistent asymptomatic intraocular inflammation [80]. In addition, Maria and colleagues [78] indicated that an increased aqueous humour flare is associated with a higher risk of developing cystic macular edema. Our study showed a higher preoperative aqueous humour flare in the OAG_c_ group than in the control group. After cataract surgery, aqueous humour flare was significantly higher in the control group compared to the preoperative result. Aqueous humour flare did not differ pre– and postoperatively in the OAG_c_ and OAG_u_ groups. De Maria and colleagues [80] found moderately lower preoperative aqueous humour flare compared to the results of our study and this may be explained by the fact that the researchers did not include glaucoma patients in their previous study.

There are important considerations of our study, including the use of topical hypotensive medications. Previously, Kahloun et al. [81] excluded participants who were treated with topical prostaglandins because prostaglandin analogues can alter the blood–aqueous barrier [83,84,85]. Arcieri and co-authors [83] investigated aqueous humour flare 4 weeks after prostaglandin analogue prescription, but did not find a significant aqueous humour flare increase. We did not exclude participants treated with prostaglandins; however, our results did not differ from Kahloun et al.’s findings [81]. Most of the participants who received antiglaucomatous therapy received a prostaglandin analogue. We also found a moderate positive correlation between the number of different antiglaucomatous medications and the aqueous humour flare value. This may indicate that although prostaglandins increase aqueous humour flare, the influence is not isolated.

Our study has some limitations, including a relatively small sample; however, we were able to reduce confounding bias in the groups via age- and gender-matching. In addition, our pilot sample of Caucasian subjects may not be generalized to different populations of patients, especially those of African descent. Additionally, information on the condition of the neck vasculature and the use of antihypertensive drugs were not included in this study, which has the potential to influence the results of the OCT-A imaging.

## 5. Conclusions

To the best of our knowledge, this is the first study evaluating the changes in IOP, total macular and RNFL thickness, ganglion cell layer, choroid, and FAZ parameters, and aqueous humour flare in OAG patients before and 6 months after cataract surgery. The IOP decreased in patients with and without OAG 6 months after cataract surgery while changes in IOP after cataract surgery were strongly negatively correlated with preoperative IOP. We did not find cystic macular edema in any of the participants 6 months after uneventful cataract surgery; however, an increase in macular thickness was observed 6 months postoperatively in all groups. Aqueous humour flare did not differ in the OAG_c_ and OAG_u_ groups before and after the surgery, but it was significantly higher in the control group compared to the preoperative result.

## Figures and Tables

**Table 1:** Demographic data of the subjects.

	Control Group (*n* = 60)	OAG_c_ Group (*n* = 40)	OAG_u_ Group (*n* = 20)	*p*
Gender male (*n*, %)	*n* = 24, 40.0%	*n* = 16, 40.0%	*n* = 8, 40.0%	>0.05
Age, mean (SD), min and max	74.03 (6.4) (59–88)	73.55 (6.6) (58–86)	73.25 (6.9) (58–86)	>0.05
Number of eyes	60	40	20	-

**Table 2:** Distribution of glaucoma medications in groups.

	OAG_c_ Group (*n* = 40)	OAG_u_ Group (*n* = 20)	Control Group (*n* = 60)	*p*
Antiglaucomatous compounds per day				* Χ^2^ = 25.69, df = 3, ***p* < 0.001**
0	0	0	60	
1	14	0	0	
2	17	3	0	
3	4	5	0	
4	5	12	0	
Antiglaucomatous medication bottles per day				* Χ^2^ = 25.02, df = 2, ***p* < 0.001**
0	0	0	60	
1	28	2	0	
2	9	8	0	
3	3	10	0	
Prostaglandin analogues (%)	77.50% (*n* = 31)	85.00% (*n* = 17)	0% (*n* = 0)	* *p* = 0.734
Beta blockers (%)	70.00% (*n* = 28)	100% (*n* = 20)	0% (*n* = 0)	* ***p* = 0.005**
Alfa-2 agonists (%)	20.00% (*n* = 8)	75% (*n* = 15)	0% (*n* = 0)	* ***p* < 0.001**
Carbonic anhydrase inhibitors (%)	27.50% (*n* = 11)	90.00% (*n* = 18)	0% (*n* = 0)	* ***p* < 0.001**
Artificial tears using ratio (%)	35.00% (*n* = 14)	35.00% (*n* = 7)	16.60% (*n* = 10)	Χ^2^ = 5.263, df = 2, *p* = 0.081
Total number of drops per day (SD)	2.7 (1.7)	4.7 (1.6)	0.4 (0.8)	***p* < 0.001**

Asterisks (*) mark data where only the OAG_c_ and OAG_u_ groups were compared as the control group was initially different. Statistically significant measurements are marked in bold.

**Table 3:** IOP changes 6 months after cataract surgery.

Group	IOP	Minimum Value	Maximum Value	Mean	SD
OAG_c_ group	Before surgery	12.0	20.0	15.8	2.1
	After surgery	7.0	16.0	12.1	2.0
	IOP change	−7.0	0	−3.7	1.7
OAG_u_ group	Before surgery	21.0	28.0	24.4	3.3
	After surgery	10.0	22.0	14.6	3.6
	IOP change	−16.0	−2.0	−8.9	4.2
Control group	Before surgery	11.0	20.0	14.9	2.4
	After surgery	7.0	17.0	13.1	2.0
	IOP change	−7.0	3.0	−1.5	2.1

## Data Availability

The data are available from the corresponding author upon reasonable request.
